# When outcome is a balance: methods to measure combined utility for the choice between induction of labour and expectant management in mild risk pregnancy at term

**DOI:** 10.1186/1471-2393-7-10

**Published:** 2007-07-04

**Authors:** Denise Bijlenga, Erwin Birnie, Ben WJ Mol, Gouke J Bonsel

**Affiliations:** 1 Department of Social Medicine, Academic Medical Centre – University of Amsterdam, PO Box 22660, NL – 1100 DD Amsterdam, The Netherlands; 2 Institute of Health Policy and Management, Erasmus MC, PO Box 1738, NL – 3000 DR Rotterdam, The Netherlands; 3 Department of Obstetrics and Gynaecology, Maxima Medical Centre, PO Box 7777, NL – 5500 MB Veldhoven, The Netherlands

## Abstract

**Background:**

When the primary and secondary outcomes of clinical studies yield ambiguous or conflicting recommendations, preference or valuation studies may help to overcome the decision problem. The present preference study is attached to two clinical studies (DIGTAT, ISRCT10363217; HYPITAT, ISRCT08132825) that evaluate induction of labour versus expectant management in term pregnancies with a mild risk profile. The purpose of the present study is to compare four methods of valuation/preference measurement.

**Methods:**

Multidimensional health state descriptions ('vignettes') defined by attributes and levels are presented to different response groups: laypersons, (ex-) patients, and medical experts. Valuations/preferences are measured with the Visual Analogue Scale (VAS), Time Trade-Off (TTO), Willingness to Pay (WTP) and Discrete Choice Experiment (DCE) techniques. These methods are compared in terms of feasibility, reliability and validity.

**Anticipated results:**

By comparing the four techniques, we aim to answer (1) which of the techniques is most feasible, reliable and valid for use in multidimensional decision problems; (2) which of the techniques can be recommended for use in economic evaluations, and (3) do different response groups produce systematically different valuations, and if so, how can these be used to interpret preference results and to contribute to the development of clinical guidelines.

## Background

Clinical trials often produce mixed results in which the primary and secondary outcomes are conflicting. Such results are difficult to transform into straightforward treatment recommendations or patient information. To overcome this decision problem, additional decision analytic strategies can be applied. One strategy is to measure disease-specific or generic health-related Quality of Life [1]. An alternative strategy is to measure explicitly the patient's (and/or expert's) preferences regarding the interventions and the full set of consequences following each of the interventions. A typical preference study derives weights on real life health states and the relevant aspects and consequences of each of the decisions based on valuation or choice experiments.

Situations in which outcomes of clinical studies are conflicting often arise in the field of obstetrics. Although often treated as a single unit, mother and child are two entities that may have conflicting interests, thus complicating the choice between alternative treatments. For example, in a pregnancy complicated by fetal growth retardation at term, theoretically it is better for the baby to be delivered. Induced delivery, however, is thought to increase the risk of requiring a caesarean section, thus harming the mother. Information on the (possible) outcomes alone is insufficient, since a clinical decision has to be made – which implies the weighing of relevant outcomes.

The present study involves two clinical dilemmas common in the third trimester of pregnancy: pregnancy-induced hypertension or pre-eclampsia (HYPITAT, ISRCT08132825) and suspected growth retardation (DIGTAT, ISRCT10363217). Whenever one of these complications occurs, a choice has to be made between either expectant management and induction of labour. Induction of labour can result in a lower risk of pregnancy complications and intrauterine death, but a higher risk of prematurity, assisted vaginal delivery and caesarean section. Expectant management involves the reverse. This choice is difficult for both physicians and parents because of the multidimensionality of the alternative treatment strategies. For instance, at least two people rather than one person bear the weight of the outcome – both mother and child. Also, the usual outcome risk is a mix of rare but very severe outcomes (e.g. mortality) and frequent but moderately important procedural and clinical outcomes. Finally, the time axis of occurrence and impact differs considerably between temporary short term and long-term health profiles.

The main objective of the present study is to compare different existing methodologies in the field of preference and utility measurement in order to arrive at a method that is feasible, reliable and valid for the analysis of multidimensional outcomes. The comparisons will be between 'attitude'-based methods that measure people's valuation of specific health states, such as the Visual Analogue Scale (VAS), Time Trade-Off (TTO) and Willingness To Pay (WTP), and 'preference'-based methods that involves a choice between two alternative health states, such as Discrete Choice Experiment (DCE). The methods have been well-described, but as yet a head-to-head comparison in this context has not been made [2-5].

## Methods

### Measurement techniques

Four candidate methodologies from different scientific backgrounds are available, all with established records of usage in clinical decision-making.

The first method is the 'attitude'-based method developed in the field of psychology [6], which applies numerical rating scales or substitutes like a VAS scale or a thermometer and sophisticated analysis to cover personal effects and error.

The second and third methods involve indirect trade-off measurements, and have specific health economic origin [7]; multiple alternative health states are valued by offering one alternative to be rated on an artificial scale. Respondents are invited to trade-off some valuable commodity in order to avoid suboptimal health or achieve optimal health. In the case of Time Trade-Off (TTO), the respondent is invited to express his/her willingness to give up part of a predefined amount of lifetime (with a maximum of 10 years) in order to avoid the presented (suboptimal) health state. In the case of Willingness to Pay (WTP), the respondent is asked to state the maximum amount of money he or she would pay to avoid the presented (suboptimal) health state.

The final measurement method is the direct trade-off method Discrete Choice Experiment (DCE; or following its analysis method also known as 'Conjoint Analysis') which has its origin in marketing research [8]. In DCE, a series of alternative non-dominant health states are presented and respondents are invited to choose one alternative from each pair. Analysis of responses can reveal the implicit weights assigned to separate attributes, and to the distinguished levels of the attributes. The DCE approach has recently gained interest among health economists but rests on a number of assumptions that may be questionable from a medical and psychological point of view.

### Stimuli

In our study the respondents are asked to value (TTO, WTP) or rank (VAS, DCE) alternative health states descriptions or 'vignettes'. The vignettes contain both a written and a visual description of the health states. The health states are defined by the dimensions ('attributes') and the level of the attributes. Attributes and levels are defined determined through open-end interviews among patients and physicians and by research data from the trial databases and the Dutch Perinatal Registry (PRN).

### Setting

Three study involves three groups of respondents; obstetrical professionals, patients/ex-patients, and lay people. The valuations and choices in all three study groups are elicited in panel sessions and by low-threshold follow-up questionnaires.

### Strategy

#### Construction of vignettes

Empirical data on the maternal and neonatal outcomes (mean, variance, range) are derived from published literature, ongoing research, and the DIGITAT and HYPITAT trials. A limited number of relevant dimensions are selected, based on existing literature, research proposals, our own research, and interviews with patients and doctors. These dimensions are transformed into attributes. The levels are chosen according to trial data and data from the PRN. Several typical cases, varying by attributes and levels, are then composed into vignettes.

#### Specification of response methods

For the 'attitude' approach, the 100-point vertical VAS is used, for indirect trade-off, a 10-year TTO and an open-end WTP are used, for the direct method the binary choice-set DCE is used.

#### Selection of respondents

Patients participating in the previously mentioned studies, caregivers (obstetricians, midwives) and laypersons. Samples are in the order of 30 patients, 100 lay people and 20 obstetrical professionals.

Ethical approval for this study was not deemed necessary by the local research council.

### Research questions

The specific research questions are as follows:

1. Which of three methods produces the best data as judged by technical failure, non-response, and inconsistencies?

2. Which method is the most reliable?

3. Which method best accounts for inter-individual heterogeneity?

4. Do preferences on the key components of the described decisions differ according to the respondents' medical experience and professional status?

5. Which method is most adequate for economic evaluation?

6. When comparing WTP with DCE including a financial domain, which economic method is more valid, reliable and feasible?

7. [On the applicational level] Which design/hypothesis testing approach appears most appropriate for this prototypical problem; if possible, what approach to sample size calculation can be derived from the answers to questions 1 to 4?

8. [On the applicational level] Which values should pregnants/patients/parents-to-be be asked for in scenarios involving a choice between induction of labour and expectant management?

### Analysis

Analysis will follow rules applicable to the four methods. The methods will be compared in terms of feasibility, reliability and validity.

## Deliverables

By rigorous comparison of the four methods, we address three questions. First, which of the four techniques is most feasible, reliable and valid for use in studies with multidimensional outcomes? Second, which of the four techniques can be recommended for use in economic evaluations? Third, do lay people, patients, and medical experts produce systematically different answers, and if so, how can these be used to interpret preference results and to contribute to the development of clinical guidelines?

## Discussion

By comparing the four preference measurement methods, and by comparing preferences between different groups of respondents, we gain insight into decision problems that sometimes arise in obstetrics. Our findings may result in a (shared) decision model and the development of clinical guidelines in obstetrics.

## Abbreviations

DIGITAT – Disproportionate intrauterine growth intervention trial at term

DCE – Discrete Choice Experiment

HTA – Health Technology Assessment

HYPITAT – Hypertension and pre-eclampsia intervention trial at term

PRN – Dutch Perinatal Registry

TTO – Time Trade-Off

VAS – Visual Analogue Scale

WTP – Willingness to Pay

## Competing interests

The author(s) declare that they have no competing interests.

## Authors' contributions

DB, EB and GJB participated in the design of the study. DB, EB, BWM and GJB participated in the coordination of the data collection. DB, EB, GJB participated in data analysis. All authors read and approved the final manuscript.

## Pre-publication history

The pre-publication history for this paper can be accessed here:


